# Changes in soil microbial community and activity caused by application of dimethachlor and linuron

**DOI:** 10.1038/s41598-021-91755-6

**Published:** 2021-06-17

**Authors:** Juraj Medo, Jana Maková, Janka Medová, Nikola Lipková, Renata Cinkocki, Radoslav Omelka, Soňa Javoreková

**Affiliations:** 1 Department of Microbiology, Faculty of Biotechnology and Food Sciences, Slovak University of Agriculture in Nitra, Tr. A. Hlinku 2, 949 76 Nitra, Slovakia; 2 Department of Mathematics, Constantine the Philosopher University in Nitra, Tr. A Hlinku 1, 949 74 Nitra, Slovakia; 3 Department of Botany and Genetics, Constantine the Philosopher University in Nitra, Nábrežie mládeže 91, 949 74 Nitra, Slovakia

**Keywords:** Microbial ecology, Microbial communities, Environmental microbiology

## Abstract

Soil microorganisms and their activities are essential for maintaining soil health and fertility. Microorganisms can be negatively affected by application of herbicides. Although effects of herbicides on microorganisms are widely studied, there is a lack of information for chloroacetamide herbicide dimethachlor. Thus, dimethachlor and well known linuron were applied to silty-loam luvisol and their effects on microorganisms were evaluated during112 days long laboratory assay. Dimethachlor and linuron were applied in doses 1.0 kg ha^−1^ and 0.8 kg ha^−1^ corresponding to 3.33 mg kg^−1^ and 2.66 mg kg^−1^ respectively. Also 100-fold doses were used for magnification of impacts. Linuron in 100-fold dose caused minor increase of respiration, temporal increase of soil microbial biomass, decrease of soil dehydrogenase activity, and altered microbial community. Dimethachlor in 100-fold dose significantly increased respiration; microbial biomass and decreased soil enzymatic activities. Microbial composition changed significantly, *Proteobacteria* abundance, particularly *Pseudomonas* and *Achromobacter* genera increased from 7 to 28th day. In-silico prediction of microbial gene expression by PICRUSt2 software revealed increased expression of genes related to xenobiotic degradation pathways. Evaluated characteristics of microbial community and activity were not affected by herbicides in recommended doses and the responsible use of both herbicides will not harm soil microbial community.

## Introduction

Modern agriculture depends heavily on pesticide use. Majority of the used pesticides are herbicides applied to almost all crops in conventional agriculture systems^1^. Environmental concerns^2^ as well as increased weed resistance^3^ and potential health issues^4^ are the most pronounced problems of herbicide use. Application of herbicides can significantly affect ecosystems on various trophic levels. Soil microorganisms pose the major drivers of organic matter transformation in soil. They are involved in nutrient cycles and create the base of soil fertility and health^5^. Any distortion in their complex relations can lead to reduced fertility^6^. Composition of the microbial community or its activity may be altered by the pesticide use, particularly when it is overused or repeatedly applied^7^. Pre-emergently or early post-emergently applied pesticides are introduced directly to soil surface thus they are in direct interaction with soil microorganisms and its dissipation relies mainly on microorganisms^8^. Information about reactions of microorganisms to the presence of herbicides is known for almost all used active ingredients; however, it is absent for some less common active ingredients such as dimethachlor assessed in this study.

Dimethachlor—2-chloro-N-(2,6-dimethylphenyl)-N-(2-methoxyethyl)acetamide is a selective herbicide belonging to the chloroacetamide family. It was first described in 1977^9^ and is currently registered in Europe for the control of broad leaved weeds in rapeseed under the trademark Teridox or in a mixture with other active ingredients as Colzor Trio (dimethachlor + napropamide + clomazone). It inhibits synthesis of very long chain fatty acids and thereby cell division. It is up-taken by weed germs, roots or cotyledons and acts mainly as a germination inhibitor. Dimethachlor has a long-term residual effect in soil and its use is restricted to rapeseed with maximum single application per 3 years^10^. The half-life of dimethachlor in soil is between 2 and 16 days^11^. However, in specific soil type and conditions half-life may be longer than 50 days^12^. Its effect on soil microorganisms is less studied in comparison with other chloroacetamide herbicides such as metolachlor or acetochlor.

Linuron—(3-(3,4-dichlorophenyl)-1-methoxy-1-methylurea) is a selective phenylurea herbicide developed in the 1960s. Herbicide formulations containing linuron are used worldwide and traded under various names (Afalon, Linuron, Uranus, and others). It is used for control of various annual grass and broad-leaved weeds in a wide spectrum of agricultural crops (beans, cotton, maize, and others). Linuron herbicides are usually applied pre-emergently or early post-emergently although some formulations allow application in later stages. The herbicide has a systemic and contact effect with residual action. Its mode of action is the inhibition of photosynthesis (photosystem II). The half-life of linuron in soil is predicted in range 10–170 days with medium 48 days in field conditions^13^. In 2017, the European Union did not approve further use of herbicides containing linuron as an active ingredient because it was classified as a possible carcinogen. It is also considered to be an endocrine disruptor because of a competitive binding to androgen receptors^14^. In the USA and most of the world it is still approved for use. In comparison with dimethachlor, the effect of linuron on soil microorganisms is better described.

For complex evaluation of pesticide effects on soil microorganisms the combination of several methods is usually involved^15^. The total amount of microorganisms is often estimated as soil microbial biomass carbon or nitrogen, while microbial activity can be well monitored by respiration analysis or by measuring various enzymatic activities^16^. The composition of the microbial community is usually determined by cultivation methods, but it does not cover all microorganisms, especially non-cultivable bacteria and archaea. High throughput sequencing on the Illumina platform greatly enhanced the resolution of insight into the bacterial community as it enabled the identification of thousands of microorganisms in each sample^17^. Although pure active ingredients are sometimes applied in laboratory assays to evaluate pesticide impact, such an attitude does not reflect real conditions. Pesticides are formulated together with adjuvants that may change their toxicity and environmental impact^18^. Evaluation of commercial formulation enables capturing changes caused by auxiliary compounds^19^. Elevated doses as high as 100-fold of field rates are commonly used in laboratory assays because it allows the observation of effects which are hindered by the natural heterogeneity of soil or when changes are too subtle to be measured when recommended dose is applied^15,20,21^.

The aim of this study was to evaluate changes in the microbial community and activity in silty-loam luvisol soil after the application of linuron and dimethachlor based herbicides in laboratory conditions. Besides recommended doses, also 100-fold higher doses were used to predict reaction of soil microorganisms after overuse of herbicides. Combining several approaches allowed us to draw a more complex picture of changes in the soil microbial community after the herbicides application.

## Results

### Microbial respiration

**Figure 1:**
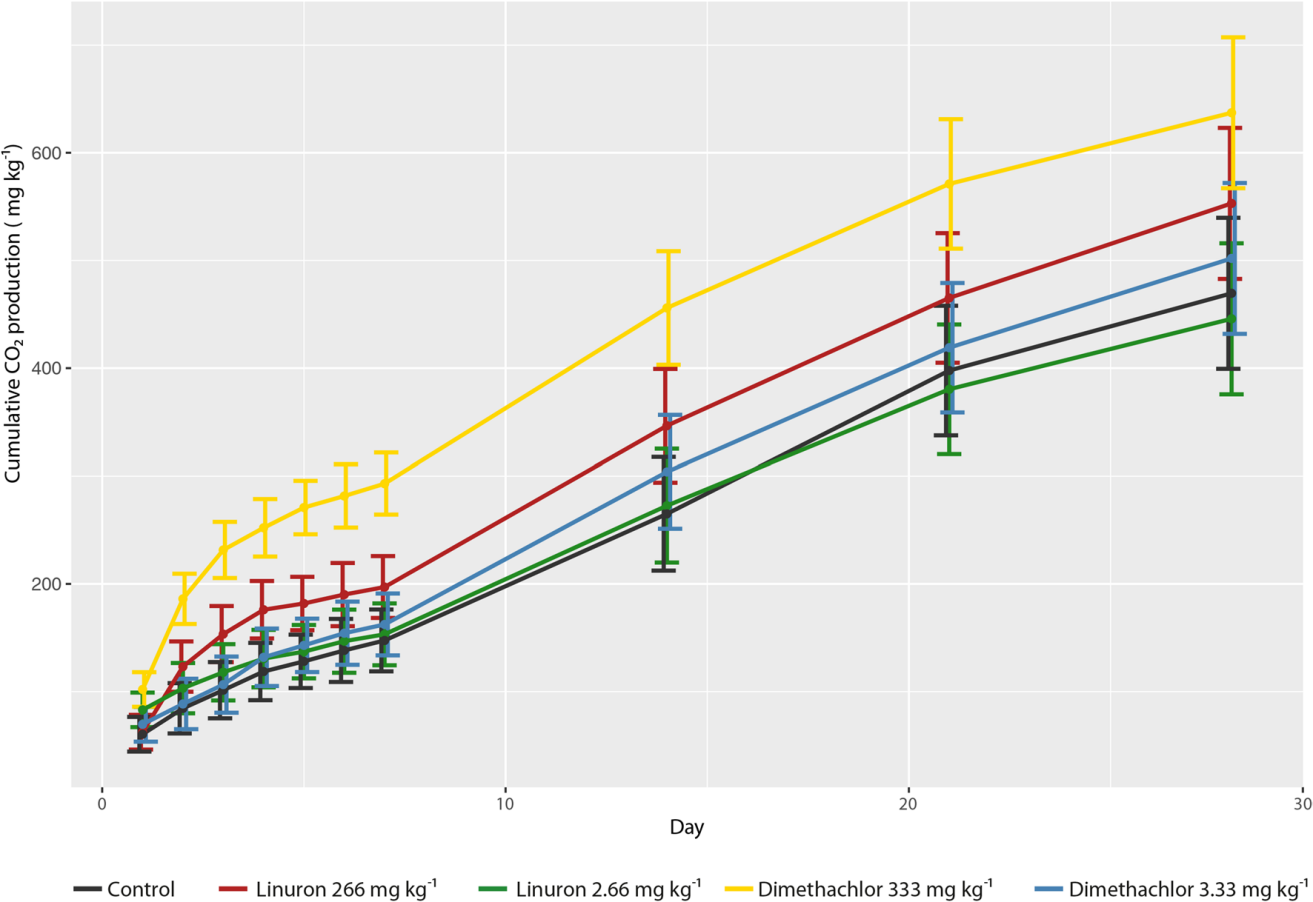
Cumulative CO_2_ production in soil treated by herbicides linuron and dimethachlor during the 28 days after treatment. In each day, values were compared by one-way ANOVA and error bars indicate Tukey confidence intervals.

Cumulative values of CO_2_ production during 28 days long incubation are summarized in Fig. 1. Respiration in soil treated by 100-fold field rate of dimethachlor significantly rose against the control during the whole observed period (ANOVA; Tukey test P < 0.05). In this treatment, CO_2_ production was 1.4 times higher than control on the 28th day. In comparison to all other treatments the respiration remains significantly higher up to 14th day. On the 21st day, the difference between 100-fold dose of dimethachlor and linuron was not statistically confirmed and at the end of assessment also respiration is soil treated by dimethachlor in recommended dose was not significantly different (ANOVA; Tukey test P > 0.05). The 100-fold dose of linuron also increased respiration however cumulative values were significantly higher than those of control only on the 4th and 5th day. In the recommended dose, herbicides did not cause a significant increase in soil respiration in comparison to control.

### Microbial biomass

**Figure 2:**
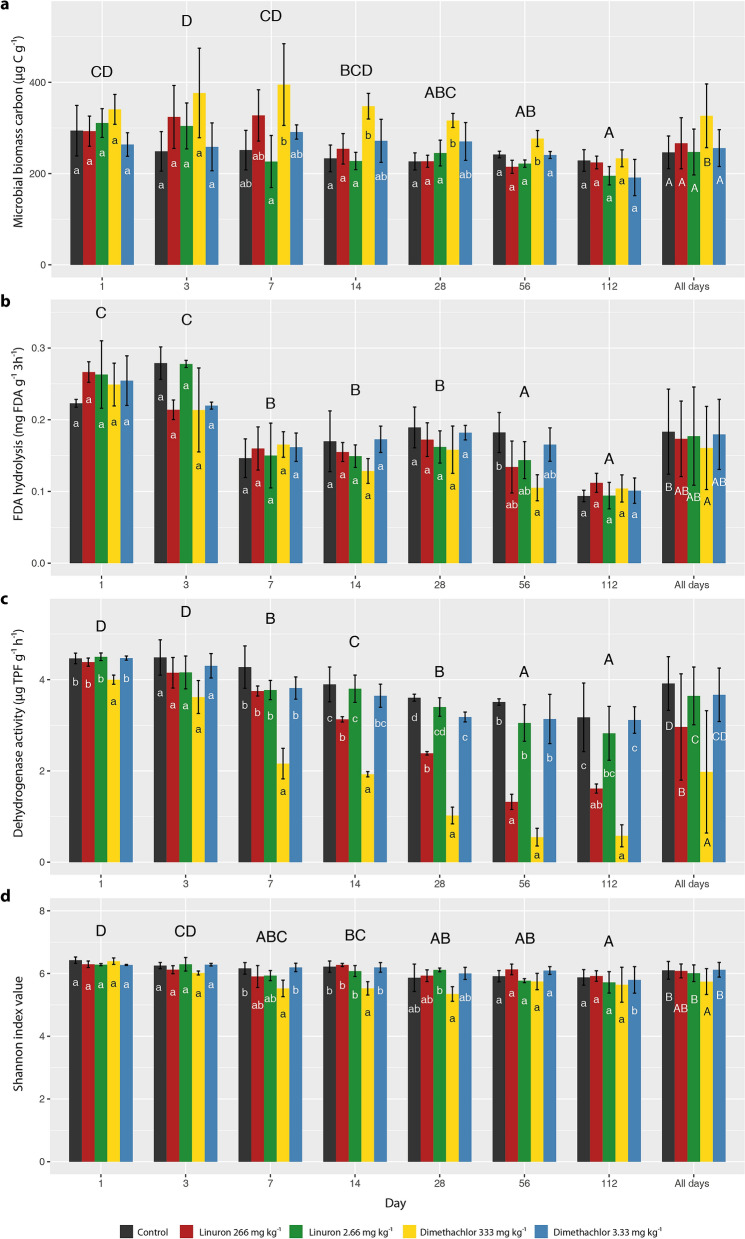
Microbial biomass carbon (**a**), Fluorescein diacetate hydrolysis (**b**) Dehydrogenase activity (**c**), and Shannon index of diversity (**d**) for soil samples treated by herbicides linuron and dimethachlor. Error bars indicate standard deviation. Columns with the same letter are not statistically different at α = 0.05 (Tukey test). Uppercase letters above days and letters in “all days” indicate differences among sampling days and treatment evaluated by two-way ANOVA with interactions. Lowercase letters indicate differences within the same day compared by one-way ANOVA.

Microbial biomass carbon was affected by length of incubation as the values gradually declined to the top in the 3rd day toward the minimum in the 112th day (Fig. 2a). The effect of treatments was significant only when high doses of dimethachlor were applied. In this treatment microbial biomass carbon increased by almost 100 µg g^−1^ during the first 7 days and then the values decreased to the same level as in other treatments on the 112th day. Significant increase in Cmic values compared to the control and linuron treatments was confirmed from the 14th to 56th day.

### Enzymatic activity

The applied herbicides affected soil enzymatic activity. FDA hydrolysis was significantly lower in dimethachlor 100-fold treatment than in control when all time-points were evaluated together (Fig. 2b). However, the difference was not confirmed in distinct time-points except for the 56th day.

Dehydrogenase activity was negatively affected by high doses of both herbicides (Fig. 2c). The high doses of dimethachlor adversely affected DHA in all days except the 3rd and high doses of Linuron significantly decreased values from the 14th day. The effect was most apparent on the 56th day when values dropped to one tenth in case of dimethachlor or to 40% in case of linuron. When both herbicides were applied in field rate, DHA was not significantly lower compared to the control. Like the microbial biomass carbon, the amounts of both enzymes showed a significant decrease during the incubation period of soil samples.

### Composition of microbiome

**Figure 3:**
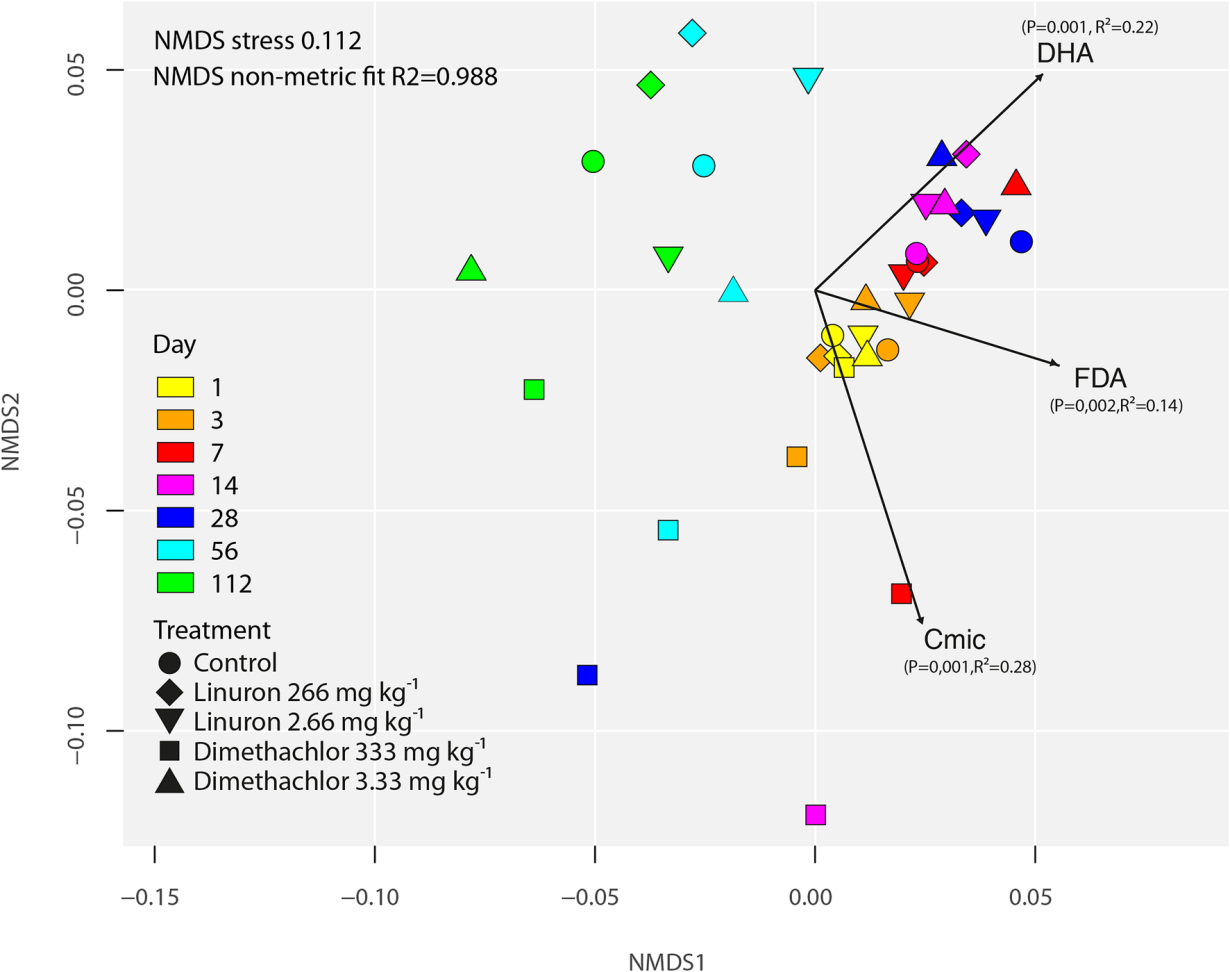
NMDS analysis of soil samples treated by herbicides linuron and dimethachlor. Arrows indicate vectors fitting for microbial biomass carbon Cmic, Fluorescein diacetate hydrolysis FDA, and Dehydrogenase activity DHA.

Together 592,143 high quality sequences were acquired by high throughput sequencing, representing 5644 sequences per replication on average. Out of them, the DADA2 algorithm generated 944 ASVs. Analysis of the data revealed significant shifts in microbiome compositions during incubation and also significant influence of treatments (PERMANOVA of Weighted Unifrac distances: day P = 0.001; treatment P = 0.002; interaction P = 0.345). However, the effect of treatments was significant only when high doses were applied. The effect of dimethachlor application was the most noticeable while compositions of soil microbiomes in other treatments were similar to that of untreated control, i.e. their change was driven primarily by the length of incubation (Fig. 3). Changes in dimethachlor treated soil became visible on the 3rd day and then were most expressed during the 7th, 14th and 28th day. In the 56th and 112th day microbiomes in these soil samples appeared to be partially recovered and tended to be similar to other treatments. Correlation of microbiome composition with soil biomass and enzymatic activities was highly significant for all parameters (Envifit, Cmic P = 0.001; DHA P = 0.001; FDA P = 0.002). Shannon’s diversity index of all treatments decreased slightly in time and significantly fell in samples treated by dimethachlor in 100-fold field rate (Fig. 2d).

Analysis of differently abundant groups of bacteria identified only a few significantly different groups of bacteria in low dose treated soils (Table 1). Of the 1416 microbial groups at all taxonomic levels (Phylum to ASV) only 27 and 33 significantly varied after linuron and dimethachlor treatments at field rate, respectively. However, 4 out of 18 phyla; 6 of 47 classes, 5 of 70 orders, 11 of 121 families, 14 of 216 genera, and 26 of 944 ASVs were differently abundant in soil treated by linuron at high dose in comparison to control soil. Difference after high dose dimethachlor application was even sharper with 11 different phyla, 18 classes, 24 orders, 30 families, 44 genera, and 96 ASVs.

**Table 1:** Bacterial taxa with different abundance in soil samples treated by herbicides linuron and dimethachlor.

Taxonomy level					Average percentage in treated soil				
Phylum	Class	Order	Family	Genus	Control	Linuron 266 mg kg^−1^	Linuron 2.66 mg kg	Dimethachlor 333 mg kg^−1^	Dimethachlor 3.33 mg kg^−1^
*Acidobacteria*					23.66	24.69	25.56	17.60***	24.13
	Gp6 group				12.34	12.25	13.45	7.80***	12.51
*Actinobacteria*					21.26	22.18	20.20	20.54	20.41
				*Gaiella*	4.67	3.84	4.31	3.56*	4.10
*Bacteroidetes*					2.99	2.86	3.30	2.16*	3.30
			*Chitinophagaceae*		2.34	2.29	2.67	1.73*	2.54
Candidate division WPS-1					7.27	7.16	8.61	5.12*	7.93
*Gemmatimonadetes*					2.01	2.37*	1.98	1.25***	2.00
*Planctomycetes*					5.95	6.57	5.40	4.51*	6.98
*Proteobacteria*					24.54	21.30*	23.38	40.06***	22.25
	*Alphaproteobacteria*				14.12	12.23*	13.11	15.26	12.27
		*Rhizobiales*			5.34	4.59	4.78	3.80***	4.77
		*Rhodospirillales*			4.20	3.44*	3.86	4.45	3.85
				*Sphingomonas*	3.16	2.25*	2.98	2.44	2.40*
				*Sphingopyxis*	0.99	1.51	1.06	2.46*	0.92
	*Betaproteobacteria*				4.46	4.56	4.69	11.32***	4.71
				*Achromobacter*	0.22	0.06	0.08	5.18***	0.06
				*Cupriavidus*	0.43	0.14	0.02*	2.29***	0.16
	*Deltaproteobacteria*				2.05	1.61*	2.52	0.98***	2.20
	*Gammaproteobacteria*				3.91	2.90**	3.05	12.50***	3.06
				*Pseudomonas*	0.44	0.28	0.34	5.53***	0.24
		*Xanthomonadales*			3.34	2.44***	2.64	6.12***	2.60
			*Xanthomonadaceae*		2.03	1.33*	1.39*	5.10***	1.39
				*Luteibacter*	0.43	0.02	0.18	3.73***	0.10
*Verrucomicrobia*					7.56	6.55	6.93	4.00***	8.13
	*Spartobacteria*				6.05	5.13	5.52	3.42***	6.21

Only groups with abundance higher than 2% in any treatment are shown. Differently abundant groups are marked (Wilcox sum-rank test, *P < 0.05, **P < 0.01, ***P < 0.001).

**Figure 4:**
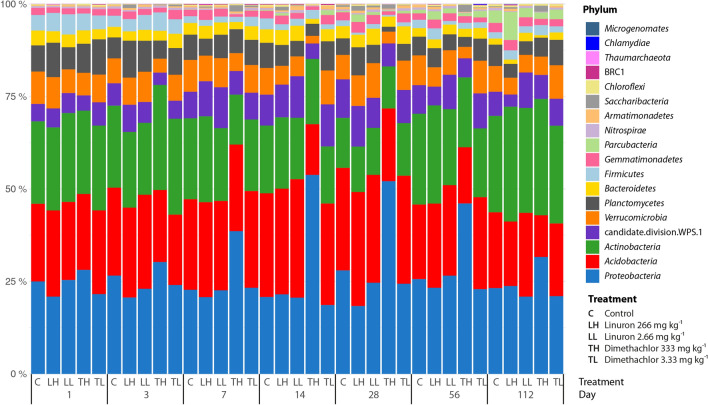
Distribution of prokaryotic phyla in soil samples treated by herbicides linuron and dimethachlor.

**Figure 5:**
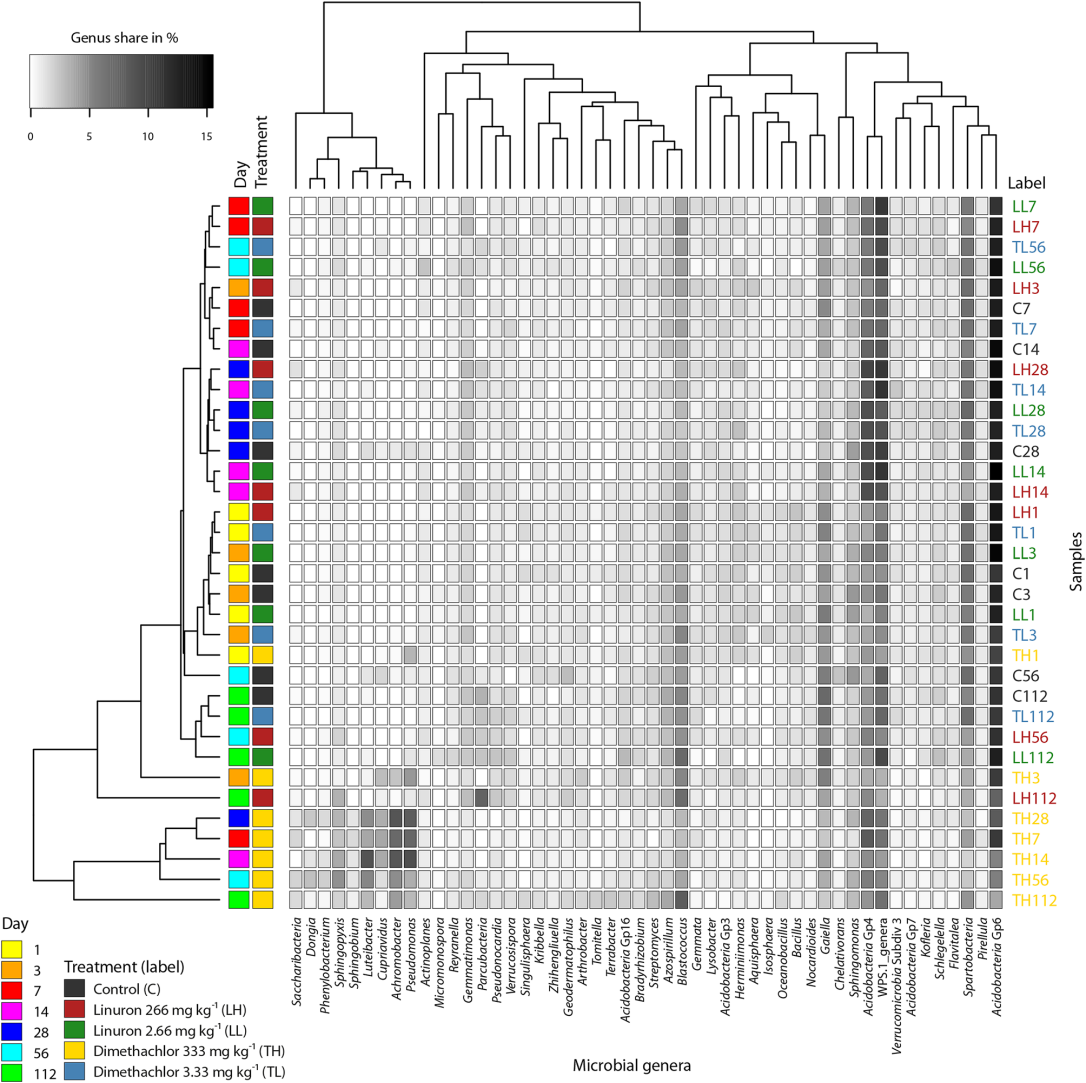
Heatmap of most common bacterial genera in soil samples treated by herbicides linuron and dimethachlor.

Differences at phylum level are visualized in Fig. 4. *Proteobacteria* and *Nitrospirae* decreased while *Gemmatimonadetes* and *Saccharibacteria* increased in linuron treated soil. Among others, the most prevalent changes caused by the dimethachlor application were the significant increase in *Proteobacteria* as well as the decrease of *Acidobacteria* (GP3; GP4; GP6; GP7). The increase of *Proteobacteria* in this group was caused mainly by increase of genus *Pseudomonas* that was ten times more abundant than in control or by *Achromobacter* that increased more than 20-fold (Fig. 5).

**Figure 6:**
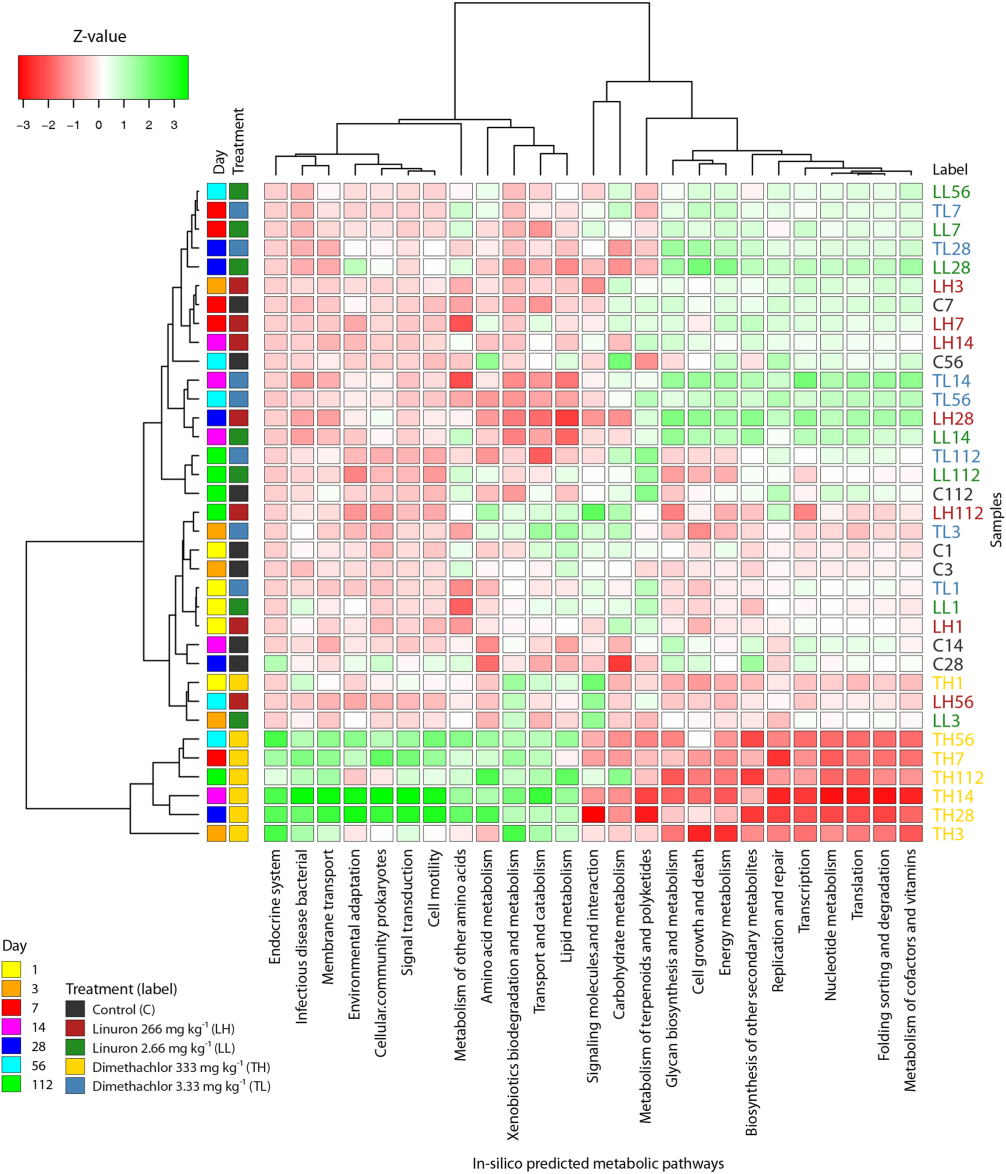
Heatmap of KEGG annotated (level 2) metabolic pathways predicted by PICRUSt2 in soil samples treated by herbicides linuron and dimethachlor. Relative expression values are column normalized and centered.

Metabolic pathways predicted by PICRUSt2 showed significant changes in the case of dimethachlor application at 100-fold field rate (Fig. 6). Of the 179 predicted pathways, 108 significantly changed their relative expression according to the DESeq2 analysis. However, the values of log_2_fold change were in fairly low range from − 0.49 to + 0.59. Up-regulation of xenobiotic degradation (caprolactam, styrene, benzoate or polycyclic aromatic hydrocarbon degradation), cell motility (bacterial chemotaxis and flagellar assembly), and membrane transport (ABC transporters and bacterial secretion system) pathways was most prevalent. In addition, some pathways from lipid metabolism such as fatty acid degradation or synthesis and degradation of ketone increased in relative predicted expression. Contrary, ansamycin, vancomycin, and streptomycin biosynthesis pathways were down-regulated probably in relation to decreased *Acidobacteria* and *Actinobacteria* abundance.

## Discussion

Introduction of pesticides into the soil environment can turn on processes which lead to promotion, inhibition or suppression of soil microbial activity^6^. Structure of the microbial community may be altered due to the proliferation of microbial groups involved in degradation or transformation of the pesticide while sensitive groups may decline. In the present study we found significant changes of microbial activity as well as significant shifts in the microbial community due to application of herbicides Teridox and linuron.

Changes in soil microbial respiration are heavily dependent on applied substance, soil type and conditions^22,23^. Decreased respiration is a sign of herbicide toxicity to the soil microbial community while increased respiration may indicate the metabolization of applied herbicide^24^. Herbicides did not reduce soil respiration and input of organic matter in field rate treatments was subtle to cause a significant increase of respiration. According to the meta-analysis of well-studied model herbicide glyphosate, soil microbial biomass or respiration are not generally affected when field rates are used^25^. The initially boosted respiration in 100-fold field rate herbicide treatments indicates faster metabolization of the dimethachlor in contrast to linuron. However, the methods used cannot resolve whether such an increase should be attributed to metabolization of active ingredients or auxiliary compounds. Pesticide adjuvants can significantly change their toxicity and environmental impact^26^. Thus, it is also important to evaluate commercial formulation rather than pure compound^19^.

In our assay, application of dimethachlor in high dose increased Cmic values. However, previous results showed that application of herbicides only exceptionally increases soil microbial biomass. Increase of biomass and respiration occurs when microorganisms use herbicide molecules as source of organic carbon, nitrogen or phosphorus, particularly in case they are deficient in soil^27^. Glyphosate significantly raised respiration and microbial biomass even in very high doses^28^. Contrary, Sulfonylurea herbicides (cinosulfuron, prosulfuron, thifensulfuron methyl, triasulfuron) increased respiration despite the decrease of biomass^29^.

Measuring of enzymatic activity is widely used in monitoring of pesticide treated soils. It indicates soil quality deterioration caused by the pollution and helps to diagnose the functional recovery of contaminated soil^30^. Depending on enzyme type, activity measurement includes contribution of enzymes in live or dead microbial cells as well as activity of extracellular enzymes or enzymes bound to soil particles (clay or humus)^24^.

Dehydrogenases are considered as strictly intracellular enzymes and their activity reflects changes in living microbial cells^31^. Dehydrogenases are oxidoreductases involved in the metabolic pathways of soil microorganisms and their activity is often used as a very sensitive marker of pesticides’ impact on soil microorganisms. Similar to our results, phenylurea herbicide alachlor decreased DHA activity significantly despite the rise in the microbial density^32^. Significant decrease of dehydrogenase activity was also observed after application of atrazine, primextra, paraquat, and glyphosate in field conditions^33^. On the other hand, only 100-fold field rate of linuron or diazinon changed DHA significantly in sandy loam and loamy sand soil^21^. Even more, Pertile et al.^34^ reported increased dehydrogenase activity following the application of imazethapyr and flumioxazin.

In our assay, dehydrogenase activity was more sensitive to application of herbicides than FDA hydrolysis when only high dose of dimethachlor decreased FDA hydrolysis. Fluorescein diacetate can be hydrolyzed by a wide spectrum of non-specific extracellular enzymes as well as membrane-bound enzymes like esterases, proteases, and lipases^35^. These enzymes are involved in microbial degradation of organic substances in soil; thus, FDA hydrolysis became a widely used estimator of microbial activity in soil, particularly after application of agrochemicals including pesticides^36,37^. Rimsulfuron, and imazethapyr caused significant changes in FDA hydrolysis either decrease or increase despite decrease in microbial biomass^38^.

Minor and transitive changes in microbial community composition after application of field rated herbicides are in accordance with results obtained by high throughput sequencing of soil treated by sulfosulfuron and chlorsulfuron^39^ or glyphosate, glufosinate, paraquat, and paraquat-diquat^40^. A significant change of microbial community was observed after application of the high dose of dimethachlor. Similar decreased diversity and distinct community in case of use 100-fold field rate of clomazone was reported^41^. Most prevalent changes were observed in the *Proteobacteria* phylum. Increase of *Proteobacteria* after application of chloroacetanilide herbicides in lab-scale wetland simulation system was found also by Elsayed^42^.

Strains from *Pseudomonas*, *Achromobacter* or *Stenotrophomonas* genera which proliferated in presence of dimethachlor were previously reported to be able to degrade various acetanilide herbicides like alachlor^43^, acetochlor^44^, butolachlor^45^. Microorganisms capable of degrading herbicide are commonly also hyper-tolerant to this herbicide^46^ which suggest finding dimethachlor degraders among groups which proliferated. In silico prediction of gene expression also supports this hypothesis. Besides the stimulation of adapted herbicide-degrading microorganisms, similar microbial responses can be explained by the activity of resistant heterotrophic microbial groups promoted by dead biomass from sensitive organisms^15^.

Changes in linuron treated soil were less apparent than in dimethachlor and no one group proliferates similarly to *Pseudomonas* in dimethachlor. Whole phylum *Proteobacteria* even decreased. Previously published phenylurea degradation-capable genera such as *Variovorax*^47^, *Sphingomonas*^48^ or *Stenotrophomonas*^49^ did not significantly increase their occurrence, either in high nor in low dose treatment. Degradation of linuron in soil is probably based on microbial consortia^50^ where initial degradation to 3,4-dichloroaniline and its further decomposition is driven by different microbial species. Degradation can be slow and may not be coupled with increased abundance of genera involved in the process.

We revealed significant changes in soil microbial characteristics only if herbicides were applied in 100-fold of field rate. Herbicides applied in recommended doses typically do not disturb microbial community and activity; and if so, their effects are transitive. However, repeated use, overdosing, and occasional spills can introduce considerable high amounts of herbicides to soil. Moreover, in effort to extrapolate the true effect of pesticide on soil microbial characteristics, scientists usually use unrealistic doses (10 or 100-fold field rates) in laboratory assays^15,20,21,36–38^. In such conditions, significant changes of enzymatic activity, biomass or community can be observed. In our case, increased dose helps us to identify microbial groups and processes probably involved in degradation of under-researched herbicidal compound dimethachlor, the active ingredient of dimethachlor. Hypothesis about metabolization of herbicides needs to be confirmed by isolation and cultivation of particular strains followed by targeted degradation study. Moreover, other methods such as stable isotope probing assays and mass spectrometry may be used for detailed insight into the fate of these compounds in soil and role of soil microorganisms in their dissipation.

Hundred-fold field rates caused shifts in microbial composition, reduced enzymatic activity but, on the other hand, increased respiration and microbial biomass. These changes were relatively small and transitory in the case of linuron but they were highly prevalent following the dimethachlor application. The application of pesticides dimethachlor and linuron at field rates neither shifted microbial community nor changed microbial activity in silty-loam luvisol during 112 days after application. Based on our results, field use of the investigated herbicides in recommended doses should not adversely affect soil microorganisms and their activities.

## Materials and methods

Laboratory experiments in controlled conditions were carried out in an effort to analyze changes in particular microbe-related characteristics in soil following the herbicide treatment.

### Soil

The soil was acquired from the experimental base of Slovak University of Agriculture in Nitra (locality Malanta, 48°19ʹ25.0″N, 18°09ʹ13.4″E, Slovakia) during June 2017. Winter wheat was grown on the experimental plot during the 2015/2016 season and maize was sown in May 2017. Analyzed herbicides had not been used in the experimental site for at least 5 years and there were not applied any herbicides during years 2016 and 2017. Soil was taken by sterilized spade at nine points arranged in W shape within a 50 × 20 m experimental plot. Each sub-sample (approx. 4 kg) taken from 0 to 20 cm depth was stored in a sterile polyethylene bag. In the laboratory, the sub-samples were mixed and sieved through a 2 mm sieve. Subsequently, the soil was pre-incubated for 1 week at 4 °C to ensure homogeneity. The detailed physical and chemical characteristics are presented in Table 2. Prior the laboratory experiments, the actual soil moisture and maximum water capacity were determined^51^.

**Table 2:** Parameters of soil used in the analysis of herbicide laboratory experiment.

Parameter	Value
Soil type	Luvisol
pH (H_2_O)/(KCl)	7.10/5.92
Bulk density	1374 kg m^−3^
Organic carbon	1.23%
Hot-water extractable carbon	402.50 µg g^−1^
Total nitrogen	1.521 g kg^−1^
Sand	21.83%
Silt	66.39%
Clay	11.78%

### Herbicides

Commercially available herbicide formulations Linurex 50 SC (Adama Ltd) with the active substance linuron (500 g l^−1^) and Teridox 500 EC (Syngenta Ltd) with the active substance dimethachlor (500 g l^−1^) were used in this study.

In the laboratory assays the herbicides were applied at either field rate or 100-fold rate. The field rate corresponded to the manufacturers’ recommended dose i. e. 1.6 l ha^−1^ for Linurex (0.8 kg ha^−1^ of linuron) and 2.0 l ha^−1^ for Teridox (1.0 kg ha^−1^ of dimethachlor). We calculated the doses of active ingredients per kg of soil assuming a homogeneous distribution of the herbicides to the depth of 2 cm and bulk density 1.5 g cm^−3^ after wetting to 40% of soil water holding capacity. The field rate Linurex treated soil (LL) contained 2.66 mg kg^−1^ of linuron while 100-fold field rate (LH) contained 266 mg kg^−1^. Soil treated by Teridox at field rate (TL) contained 3.33 mg kg^−1^ of dimethachlor and 100-fold field rate (TH) contained 333 mg kg^−1^. No herbicide treated control (C) was also included. All experiments were conducted at 40% of soil water holding capacity. The required amount of water was calculated according to the soil water holding capacity, soil weight, and actual soil water content. The herbicides were diluted in a calculated amount of sterile deionized water to achieve the selected dose and applied by hand sprayer on the top of the soil. Only sterile water was used for the control.

### Laboratory assay

To evaluate changes in soil microbial biomass, enzymatic activity and microbial community composition, the soil was incubated in plastic boxes under laboratory conditions. The design of the laboratory assay was similar to that described in our previous work^20,39^. Each plastic box (approx. 18 × 6 × 12 cm) was filled with 350 g of soil in an approx. 2 cm layer. Together 105 boxes were prepared to achieve the saturated 2-factorial design combining 5 variants, and 7 independent time measurements with 3 replications. Boxes were covered with perforated lids and incubated in the dark at 26 °C. Soil was moistened with sterile water once a week to maintain constant moisture. An effect of herbicides on microbial parameters was analyzed in 7 time-points, on the 1st, 3rd, 7th, 14th, 28th, 56th, and 112th after application of herbicides. Before the measurement, the soil in each box was thoroughly mixed.

### Microbial respiration assay

A respiration assay for all five treatments was set up separately, using the same doses and applications of herbicides as described previously, but the amount of the soil in each sample was 50 g. Microbial respiration was measured according to Alef^52^ as CO_2_ sorption in a closed system of the 1 l jar. CO_2_ was trapped in a beaker containing 0.1 M KOH, and residual KOH was titrated by 0.1 M HCl. Three jars (replications) were prepared for each treatment. The amount of CO_2_ was measured every 24 h during the first 7 days and on the 14th, 21st and 28th day of incubation. The results are expressed as cumulative values of CO_2_ production over 28 days.

### Analysis of microbial community

Homogenized soil (0.25 g) from each plastic box was subjected to DNA extraction using Mobio Ultraclean soil DNA extraction kit according to manufacturer instructions. Variable regions V3 and V4 of 16S rRNA gene were amplified using primers 341r and 785f^53^. Primers were enhanced by 6 bp long internal barcode coupled with 0–3 bases for library diversification which improved sequencing of low diversity libraries^54^. Amplified PCR products were purified, normalized to the same concentration, and pooled. The sequencing library was constructed using an 8-cycle PCR with NexteraXT primers with indices. The library was quantified and quality-checked by electronic electrophoresis Shimazu multiNA2. Sequencing was performed on Illumina MiSeq using V3 2 × 300 bp paired end kit.

Acquired sequences were quality filtered and samples were identified according to the barcodes using software SEED2^55^. The sequences were further processed by QIIME2^56^ and amplicon sequence variants (ASVs) were generated by DADA2 algorithm^57^. ASVs were identified against the RDP database^58^. In silico prediction of metabolic pathways was done using software “phylogenetic investigation of communities by reconstruction of unobserved states—PICRUSt2”^59^. Predicted pathways were categorized according to KEGG (Kyoto Encyclopedia of Genes and Genomes) orthology groups and compared between samples using DESeq2^60^. For comparison of diversity indices and community composition the sequences were rarefied to lowest sequence count (2997 sequences). For comparison and visualization of communities weighted Unifrac distances^61^ were calculated from aligned sequences of ASVs. The sequences were deposited in GenBank under BioProject number PRJNA685958.

### Microbial biomass carbon

Microbial biomass carbon (Cmic) was measured by fumigation-extraction method^62^. Soil extract was prepared from 10 g of moist soil from plastic box by homogenization with 40 ml of 500 mmol K_2_SO_4_ on a horizontal shaker (150 rpm) for 30 min followed by filtration. Also, 10 g of soil from the same plastic box were fumigated with ethanol-free chloroform fumes at room temperature in desiccator for 24 h and then extracted equally. In the filtrate, the carbon content was determined by the dichromate oxidation method. The amount of microbial biomass carbon was estimated as Cmic = 2.64 × EC, where EC is the difference between K_2_SO_4_ extractable carbon from the fumigated and from non-fumigated soil and 2.64 is coefficient describing relation between microbial biomass carbon and extractable carbon due to fumigation proposed by Vance et al.^62^.

### Enzymatic activity

Dehydrogenase activity (DHA) was measured according to Casida et al.^63^. Three grams of soil from the plastic box were placed into a 16-ml tube, then 1 ml of TTC (triphenyltetrazolium chloride) solution and 2.5 ml of distilled water were added to the tube. Tube was sealed and incubated at 37 °C. After 24 h, 5 ml of methanol were added for TPF (triphenyl formazan) extraction. The intensity of the reddish color of the extracts was measured with a spectrophotometer at a wavelength of 485 nm using methanol as a blank. The amount of produced TPF was calculated according to the calibration curve.

The hydrolysis of fluorescein diacetate (FDA hydrolysis) was measured using the method described by Green et al.^64^. Briefly, 1 g of soil from the plastic box was incubated in a 250 ml-Erlenmeyer flask containing 50 ml of 60 mM Na-phosphate solution (buffered at pH 7.6). The reaction was started by adding 0.5 ml of a FDA solution (2 mg ml^−1^) and incubated for 3 h in a shaker incubator at 37 °C and 200 rpm. After removing the soil by centrifugation and filtration, the hydrolyzed FDA was measured with a spectrophotometer at a wave-length of 490 nm. The concentration of released fluorescein was calculated by reference to a standard curve prepared from fluorescein standards.

### Statistical evaluation

All statistical analyses were done in the R environment^65^. Cumulative values of CO_2_ production were compared by one-way analysis of variance (ANOVA) followed by post-hoc Tukey test for each day separately. Values of Cmic, FDA, and DHA were compared by two-way ANOVA with interactions followed by Tukey test. As the values showed significant interactions, one-way ANOVA and Tukey tests were used for evaluation of differences in Cmic, FDA, and DHA in each sampling day. For all ANOVA analyzed data, normality was confirmed by Shapiro–Wilk test.

Communities were compared using permutational analysis of variance (PERMANOVA) based on weighted Unifrac distances and composition was visualized by non-metric multidimensional scaling (NMDS) using package vegan^66^. Differences in abundance of ASVs and higher taxonomic groups were tested by Wilcoxon rank-sum test.

## Data Availability

All data are available from corresponding author upon request.
